# Predictors of Costs in Dementia in a Longitudinal Perspective

**DOI:** 10.1371/journal.pone.0070018

**Published:** 2013-07-18

**Authors:** Hanna Leicht, Hans-Helmut König, Nina Stuhldreher, Cadja Bachmann, Horst Bickel, Angela Fuchs, Kathrin Heser, Frank Jessen, Mirjam Köhler, Melanie Luppa, Edelgard Mösch, Michael Pentzek, Steffi Riedel-Heller, Martin Scherer, Jochen Werle, Siegfried Weyerer, Birgitt Wiese, Wolfgang Maier, for the AgeCoDe study group

**Affiliations:** 1 Department of Health Economics and Health Services Research, Hamburg Center for Health Economics, University Medical Center Hamburg-Eppendorf, Hamburg, Germany; 2 Department of Primary Medical Care, University Medical Center Hamburg-Eppendorf, Hamburg, Germany; 3 Clinic and Policlinic for Psychiatry and Psychotherapy, Munich Technical University, Munich, Germany; 4 Department of General Practice, Medical Faculty, University of Düsseldorf, Düsseldorf, Germany; 5 Department of Psychiatry, University of Bonn, Bonn, Germany; 6 German Center for Neurodegenerative Diseases (DZNE), Bonn, Germany; 7 Institute of Social Medicine and Occupational Health, University of Leipzig, Leipzig, Germany; 8 Central Institute of Mental Health, Medical Faculty Mannheim/Heidelberg University, Mannheim, Germany; 9 Institute for Biometrics, Hannover Medical School, Hannover, Germany; Cardiff University, United States of America

## Abstract

**Objective:**

To analyse predictors of costs in dementia from a societal perspective in a longitudinal setting.

**Method:**

Healthcare resource use and costs were assessed retrospectively using a questionnaire in four waves at 6-month intervals in a sample of dementia patients (N = 175). Sociodemographic data, dementia severity and comorbidity at baseline, cognitive impairment and impairment in basic and instrumental activities of daily living were also recorded. Linear mixed regression models with random intercepts for individuals were used to analyse predictors of total and sector-specific costs.

**Results:**

Impairment in activities of daily living significantly predicted total costs in dementia patients, with associations between basic activities of daily living and formal care costs on the one and instrumental activities of daily living and informal care costs on the other hand. Nursing home residence was associated with lower total costs than residence in the community. There was no effect of cognition on total or sector-specific costs.

**Conclusion:**

Cognitive deficits in dementia are associated with costs only via their effect on the patients' capacity for activities of daily living. Transition into a nursing home may reduce total costs from a societal perspective, owing to the fact that a high amount of informal care required by severely demented patients prior to transition into a nursing home may cause higher costs than inpatient nursing care.

## Introduction

For Germany, the costs of illness (COI) of dementia in the population aged 65 and older were € 10.285 billion (US-$ 14.296 billion) in 2008, according to federal statistics [1]. This corresponded to 8.4% of all costs of illness in the same age bracket, which makes dementia one of the most expensive disease categories in old age. However, these data reflect medical and formal nursing care costs from a payer perspective, but do not include costs of care provided informally, so that the total costs of dementia in Germany from a societal perspective can be assumed to be substantially higher. Moreover, it is expected that due to demographic change expenditures associated with dementia will rise considerably in the future, especially as the baby boomer generation enters old age in the coming decades [2]. In 2010, 1.2 million dementia patients were estimated to be living in Germany, but this figure is expected to rise to 1.5 million in 2020 and to 2.6 million in 2050 [3]. Dementia as a syndrome is characterised clinically by progressive cognitive impairment which leads to increasing deficits in activities of daily living. It can be caused by different underlying diseases, the most common of which is Alzheimer's disease [4]. Dementia is associated with substantial need for care and supervision, which rises as the disease progresses. There is ample evidence to show that costs in dementia are generally characterised by three patterns: first, costs of dementia are to a large extent costs of nursing care, second, costs of nursing care increase substantially over the course of the disease (while most studies do not find an association between dementia severity and medical care costs), and third, informal care accounts for a substantial share of total costs if patients are cared for in the community (see [5] and [6] for reviews). The findings of the two most recent cross-sectional German studies which included informal care are in accordance with these patterns. Schwarzkopf et al. [7] reported total annual cost of € 40,000 (US-$ 55,600) in mild and € 62,800 (US-$ 87,290) in moderate dementia at year 2008 values for dementia patients cared for in the community. Informal care accounted for approximately 80% of total costs in this sample. In a study which analysed a mixed sample of dementia patients living in the community or in a nursing home as well as non-demented control subjects drawn from the AgeCoDe cohort, we found annual excess costs of € 15,500 in mild, € 31,600 in moderate and € 41,800 in severe dementia at year 2008 values (corresponding to US-$ 21,550, 43,920 and 58,100, respectively) [8]. Across disease stages approximately half of all costs were due to informal care.

The impact of dementia on costs of care is thus well-documented. However, virtually all of these findings are based on cross-sectional studies, while little has been published so far on the development of costs over time and its predictors. In the few longitudinal studies published so far, functional impairment was the only predictor that was invariably associated with both formal and informal care costs [9]–[13].

The current study is a longitudinal analysis of costs in dementia from a societal perspective. The data were collected as part of the AgeCoDe study, and the aim of this study is to investigate potential predictors of costs over time, including medical care costs as well as formal and informal care costs.

## Methods

### Ethics Statement

The ethics committees of the participating centers approved the study (reference numbers: 050/02 (University of Bonn), 2079 (Faculty of Medicine, University of Düsseldorf), 2817/2007 (Hamburg Medical Association), 309/2007 (Faculty of Medicine, University of Leipzig), 2007-253E-MA (Medical Ethics Commission II, University of Heidelberg at the University Medical Center of Mannheim), 713/02 (Faculty of Medicine, Technical University of Munich)). The study was conducted according to the principles expressed in the Declaration of Helsinki. Written informed consent was obtained from all participants at recruitment. Once a patient had been diagnosed with dementia, written informed consent was obtained from a proxy.

### Data and Samples

Data were collected as part of the German Study on Ageing, Cognition and Dementia in Primary Care Patients (AgeCoDe). The subjects in the AgeCoDe cohort were recruited through general practitioners' (GP) offices at six study centres throughout Germany during the baseline assessment in 2003 and 2004, and have since been followed up at 1.5-year intervals (to date, the study is ongoing). Inclusion criteria at recruitment were age 75 years and above, absence of dementia and at least one contact with the GP during the previous 12 months. Exclusion criteria at recruitment were insufficient German language skills, GP consultation by home visits only, residence in a nursing home, severe illness which the GP would deem fatal within 3 months, deafness or blindness, and lack of ability to provide informed consent. Details regarding the cohort have been published elsewhere [14].

**Figure 1:**
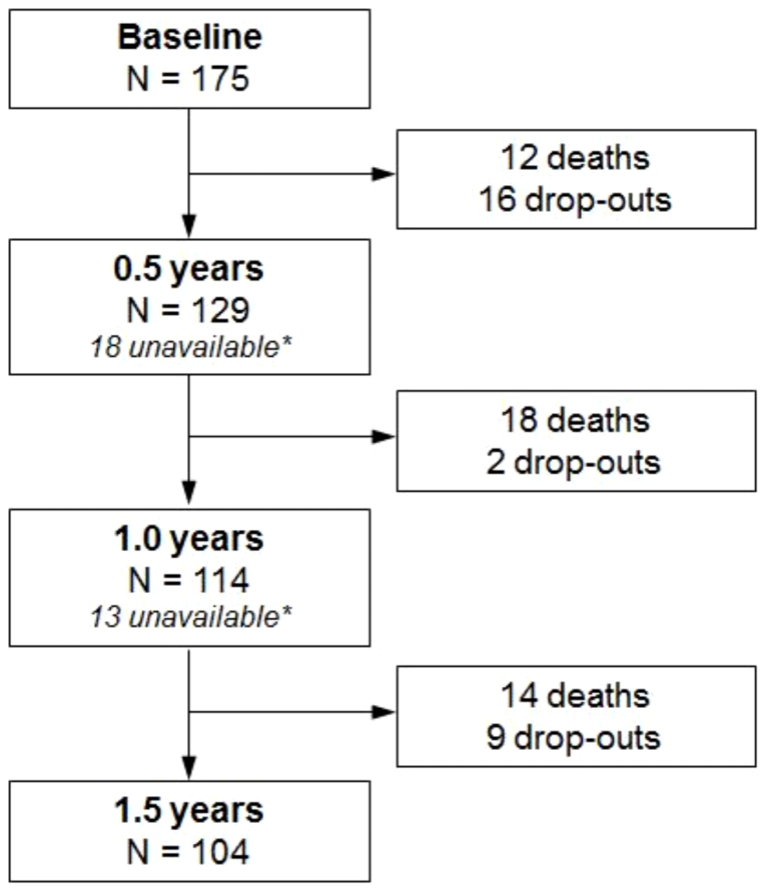
Sample sizes and drop-outs across waves. * These are patients who were unavailable for interview due to reasons such as illness for the respective assessment wave, but remained in the study and were interviewed in subsequent waves.

The data for the analyses presented here were obtained from a subsample of the AgeCoDe cohort which comprises those subjects who had received a diagnosis of dementia by the time of the third AgeCoDe follow-up wave (approximately 4.5 years after the AgeCoDe baseline; N = 175). For this subsample an assessment of healthcare resource use was introduced into the assessment battery at the third follow-up wave, and subjects were given additional follow-up assessments at 6-month intervals between the major AgeCoDe follow-up waves. The present analyses comprise healthcare resource use data from four assessments at 6-month intervals, starting with the third AgeCoDe follow-up wave and covering a time-span of 1.5 years. For the purposes of this study, the third AgeCoDe follow-up wave is henceforth regarded as the baseline. 1.5 years after baseline, 104 dementia patients remained in the sample, while 44 patients had died and 27 had dropped out for other reasons (see Figure 1). Data were collected in structured interviews with the patients and their proxies that were conducted by trained staff at the patients' homes.

### Sociodemographic and clinical variables

Sociodemographic data include age, sex, family status and living situation. Classification of dementia severity for the purposes of this study was based on the Clinical Dementia Rating (CDR [15]), which covers deficits in memory and other cognitive domains as well as impairment in activities of daily living. The CDR yields an overall score of 0 (normal), 0.5 (very mild dementia), 1 (mild dementia), 2 (moderate dementia) or 3 (severe dementia). “Mild dementia” in this study. corresponds to a CDR score of 1 or less, while “moderate” and “severe dementia” correspond to the CDR scores 2 and 3, respectively. The Mini-Mental Status Exam (MMSE [16]) and the Global Deterioration Scale (GDS [17]) were also used to assess cognitive impairment. The MMSE is a screening instrument for cognitive impairment with a total score ranging from 30 (no impairment) to 0. The GDS is used to measure dementia severity and produces a score between 1 (no impairment) and 7 (severe dementia). The Barthel Index [18] was employed to assess deficits in activities of daily living (ADL). The degree of impairment is measured indicated on a scale between 100 (no deficits) and 0. Impairment in instrumental activities of daily living (IADL), i.e. activities beyond self-care, was recorded by means of the IADL scale by Lawton and Brody [19], which produces a score between 8 (no impairment) and 0.

### Diagnosis of dementia

Dementia was diagnosed on the basis of a consensus between the interviewer and an experienced geriatrician or geriatric psychiatrist according to DSM-IV criteria for dementia of different types as implemented in the structured SIDAM interview [20]. Diagnostic criteria were objective deficits in memory and another cognitive domain as well as ADL impairment.

### Comorbidity

At the third follow-up assessment, twenty-eight chronic conditions other than dementia were scored as present or absent and rated from 1–4 according to severity if present by the subjects' GPs. These data were combined into simple and weighted count scores (weighted score: sum of severity ratings for conditions scored as present). The list of conditions includes diabetes, hypertension, cardiac arrhythmia, coronary heart disease, myocardial infarction, hyperlipidemia, hypercholesteremia, chronic heart failure, peripheral arterial disease, Parkinson's disease, epilepsy, depression, alcohol abuse, stenosis, transient ischaemic attack, stroke, hyperthyroidism, hypothyroidism, renal insufficiency, chronic liver disease, traumatic brain injury, back pain, arthrosis, obesity, gout, varicose veins, chronic obstructive pulmonary disease, asthma and gastritis. The list of comorbidities was compiled to reflect both conditions which constitute risk factors for dementia according to the literature, and conditions which are frequent in the general population of the age range investigated in this study.

### Healthcare resource use

Resource use was recorded by means of a questionnaire administered as part of the interview, using a proxy version that was completed by a caregiver. The resource use questionnaire is adapted from questionnaires used in previous investigations by the same working group (e.g. [21], [22]) and is available from the authors upon request. The questionnaire covers in-patient treatment, out-patient physician treatment, pharmaceuticals, other kinds of out-patient treatment (such as physical or occupational therapy), medical supplies and dental prostheses, nursing home care, professional nursing services and other paid help as well as informal care (see Table 1). This latter part of the questionnaire is based on an instrument developed by Neubauer et al. [23], which again is based on the Resource Utilisation in Dementia instrument [24]. It contains separate items for the number of hours per day spent by informal caregivers on basic care, shopping, housework, assistance with medication, preparation of meals, financial matters, or taking the patient to appointments etc. Hours of supervision were also included in the questionnaire, but were not evaluated in the results presented here due to lack of reliability, which is evidenced by numerous entries of “24 hours per day” for supervision, resulting in wildly implausible total hours of care per day. Assessment was retrospective and covered a period of 3 months, except for in-patient treatment and nursing-home care for which the period was 6 months. In order to minimise recall bias, the questionnaire contained lists of common resources and services.

**Table 1:** Unit costs (base case analysis).

Sector	Services/Goods	Units	Unit costs *(Source)*
Inpatient treatment	Stays in general hospitals, specialised psychiatric and neurological hospitals or rehabilitation clinics (including day-patient treatment)	Days in hospital	Per diem costs by type (Federal Statistical Office, German Hospital Federation, Statutory Pension Insurance Fund [33]–[35])
Outpatient physician treatment	Treatment by GPs, specialists and outpatient clinics	Number of contacts	Calculated costs per contact, by specialisation [36]
Other outpatient treatment	E.g., physiotherapy, massage, occupational therapy, speech therapy	Number of contacts	Reimbursement schedules (Statutory health insurance funds [37]–[39]), calculated costs per contact [36], by type
Medical supplies and dental prostheses	E.g., walkers, incontinence pads, hearing aids, surgical stockings; bridge, crown	Quantity	Reimbursement schedules (Statutory health insurance funds, Federal Association of Panel Dentists [40], [41]), calculated costs per item [34], by type
Pharmaceuticals	Specific products (including trade name, drug code, package size, pharmaceutical form, dosage)	Quantity	Pharmacy retail prices (Rote Liste 2008 [42])
Nursing home care	Residential care, day care	Days	Calculated costs of care per day (Federal Statistical Office [26]), by type
Professional home care	Care and assistance provided by professional nursing services and other paid help, differentiated by type (e.g., basic care, assistance with cleaning, shopping, financial matters etc.) and limited to care or assistance required due to illness or age	Hours	Hourly gross wage rate plus non-wage labour costs for employees in the domain of care and assistance for the elderly or handicapped (Federal Statistical Office [43], [44]): € 18.69/h
Informal care	Care and assistance provided by family or friends, differentiated by type and limited to care or assistance required due to illness or age	Hours	*Replacement cost method:* Hourly gross wage rate plus non-wage labour costs for employees in the domain of care and assistance for the elderly or handicapped (Federal Statistical Office [43], [44]): € 18.69/h

### Healthcare costs

Resource use was assessed from a societal perspective, therefore all resources and services used were recorded, regardless of whether they were paid for out-of-pocket or covered by health or nursing insurance. The costs analysed in this study are direct costs of illness, arising from the use of resources. Indirect costs due to lost productivity are disregarded due to the advanced age of the subjects.

Healthcare costs were calculated for a 6-month period, multiplying resource use by two in sections which covered a 3-month period. Costs were calculated from resource use as recorded in the questionnaire by means of unit costs, the sources of which are listed in Table 1. Informal care was valued using the replacement cost approach (or proxy good method), i.e. it was assumed that the same amount of care would have been provided by professional nursing services in the absence of an informal caregiver. Therefore, hours of informal care were valued using the same hourly wage rate as for professional home care (see van den Berg [25] for an overview of methods for the valuation of informal care). This rate was €18.69 per hour (US-$ 25.98) and reflects the average gross wage rate plus non-wage labour costs for employees in the domain of care and assistance for the elderly or handicapped. Nursing home residence was valued at rates of €56.40, €70.76 and €85.13 per day (US-$ 78.40, US-$ 98.36 and US-$ 118.33) according to care level [26]. Details regarding specific unit costs can be found in Table S1. Cost were calculated in € at 2008 price levels. Unit costs that were unavailable at year 2008 values were inflated or deflated to year 2008 price levels by means of the consumer price index [27]. For comparability, costs in € at year 2008 price levels were converted to US-$ at a rate of 1.39 US-$ per € [28].

### Sensitivity analysis

In a sensitivity analysis, the unit costs for informal care were varied using three additional approaches. Under the replacement cost approach as in the base case analysis, hours of informal care were valued using the minimum wage in health care and nursing professions which has been effective in Germany as of July 2010. As a population-weighted average of the gross hourly rates for eastern and western Germany (7.50 € and 8.50 €, respectively) plus non-wage labour costs, a rate of 10.96 €/hour was applied.

In addition, we used the opportunity cost approach under which hours of informal care are valued according to their best alternative use [25]. If informal care is assumed to constitute lost leisure time (as would apply if a patient is cared for by a spouse of retirement age or by a child in his or her spare time), this is valued at the mean hourly net wage plus unemployment and pension insurance contributions [29]. In 2008 the corresponding hourly rate for Germany was 18.00 € [30], [31] and thus almost identical to the replacement costs used in our baseline analysis. If informal care is assumed to constitute lost production in the formal economy (corresponding to a situation where a spouse or child gives up paid employment in order to provide informal care), it is valued at the mean hourly market wage rate including non-wage labour costs [25]. In 2008 the corresponding hourly rate for Germany was 29.60 € [31].

### Statistical analyses

Missing values for quantities of resource use were imputed using the means of the observed data for the respective items (conditional means), with the exception of missing values for the dosage of medication. As medications and their dosage were too varied interindividually for mean imputation to be possible, costs for medication with missing values for dosage were calculated using a conservative rule, whereby the pharmacy retail price of one package of the drug per 3 months was applied. Missing values occurred for individual items across sections, but made up no more than 2.3% of the data, except for one particular item concerning time spent by informal caregivers on financial matters, which produced up to 5.2% missing values. Between 1.6% (third follow-up) and 3.0% (baseline) of entries for medication could not be processed on account of insufficiently specific data and were excluded from the analysis.

Differences in proportions were tested by means of the χ^2^ test or Fisher's exact test, as appropriate. Group differences were analysed using two-tailed *t*-tests.

Linear mixed regression models with random intercepts for individuals were used for longitudinal analyses of the factors which determine costs in dementia patients. These analyses were calculated for total costs as well as for the subcategories medical care costs, formal care costs and informal care costs, using the data from all four waves, with the waves entered into the model as the variable “time”. The coefficient for “time” therefore corresponds to the changes in costs that occur over a six-month interval, controlling for the other predictors. Age, sex and the weighted comorbidity score at baseline were entered as time-independent variables, while nursing home residence (reference category: living at home) and the Barthel index, IADL and GDS scores were entered as time-dependent variables. In the sensitivity analysis, the same models were estimated again for informal care costs and total costs in the three different scenarios.

We used bootstrapped standard errors (based on 4,000 replications) in the regression analyses to account for the skewness of the cost data [32]. We also estimated alternative models with an additional random intercept for study center. However, as there was no significant effect for study center and these models virtually did not differ from the simpler ones, the random intercept for study center was dropped from analysis.

Statistical analysis was performed using STATA Release 11 (Stata Corp., College Station, Texas).

## Results

### Sociodemographic and clinical variables

Sociodemographic and clinical data for the sample at baseline and at 1.5 years after baseline (third follow-up) are presented in Table 2. At baseline, subjects were on average 85.3 years old, with 69% being female. Overall, the clinical variables indicate substantial impairment among the patients at baseline.

**Table 2:** Demographic and clinical variables.

	Baseline (N = 175)	1.5 years (N = 104)
**Female**: N (%)	120 (68.6)	74 (71.2)
**Age**: mean (range)	85.3 (79–96)	87.0 (81–98)
**Marital status**: N (%)^a^		
Single	13 (7.4)	9 (9.1)
Married	67 (38.3)	36 (34.6)
Divorced	4 (2.3)	0
Widowed	91 (52.0)	54 (51.2)
**Living situation**: N (%)^b^		
Alone	37 (21.1)	13 (12.5)
With spouse/partner	50 (28.6)	26 (15.0)
With other relatives	15 (8.6)	9 (8.7)
Nursing home	47 (26.9)	40 (38.5)
Assisted living	7 (4.0)	4 (3.9)
Retirement home	8 (4.6)	3 (2.9)
Other	10 (5.7)	7 (6.7)
**MMSE**: mean (range)	19.4 (0–27)^c^	17.6 (0–28)^d^
**Comorbidity**: mean (range)		
Simple count score	5.7 (0–27)	5.2 (0–16)
Weighted count score	10.2 (0–71)	8.7 (0–36)
**Barthel index**: mean (range)	69.5 (0–100)	63.0 (0–100)^e^
**IADL scale**: mean (range)	2.3 (0–8)	1.6 (0–8)^e^
**GDS**: mean (range)	4.6 (3–7)	5.0 (4–7)^e^
**Dementia severity**: N(%)		
Mild	121 (69.1)	50 (48.1)^e^
Moderate	31 (17.7)	27 (26.0)^e^
Severe	23 (13.1)	22 (21.2)^e^

MMSE  =  Mini-Mental Status Examination; IADL  =  instrumental activities of daily living; GDS  =  Global Deterioration Scale;

a 5 missing values at 1.5 years;

b 1 missing value at baseline and 2 missing values at 1.5 years;

c 22 missing values in the sample;

d 11 missing values in the sample;

e 5 missing values in the sample.

There are clear trends over time in the sample indicating lower MMSE, Barthel index, IADL and GDS scores 1.5 years after the baseline assessment. Also, the proportions of patients with mild, moderate and severe dementia had shifted toward the more severe stages, with 26.0% and 21.2% in the moderate and severe categories 1.5 years after baseline as opposed to 17.7% and 13.1% at baseline, and the proportion of nursing home residents had risen from 27.4% to 38.5%. A comparison of baseline data for subjects with complete follow-up and for those who dropped out of the cohort at any stage after the baseline assessment indicated that drop-outs were initially more severely impaired, which was evident in MMSE scores, Barthel scores, IADL scores and GDS scores (Table 3).

**Table 3:** Comparison of complete cases and drop-outs.

Baseline data	Patients with complete data (N = 104)	Drop-outs after baseline (N = 71)	*p* value
**Age**: mean(range)	85.4 (79–96)	85.2 (80–93)	0.645^a^
**Female**: N (%)	74 (71.2)	46 (64.8)	0.373^b^
**MMSE**: mean(range)	20.6 (4–26)^c^	17.4 (0–27)^c^	<0.001^a^
**Comorbidity**: mean (range)^d^	5.5 (0–21)	6.0 (0–27)	0.497^a^
**Nursing home care**: N (%)	24 (23.1)	24 (33.8)	0.118^b^
**Barthel Index**: mean(range)	75.5 (10–100)	60.8 (0–100)	0.001^a^
**IADL scale**: mean(range)	2.7 (0–8)	1.7 (0–8)	0.002^a^
**GDS**: mean(range)	4.4 (3–7)	4.9 (4–7)	<0.001^a^
**Dementia severity**: N(%)			
Mild	83 (79.1)	38 (53.5)	0.001^b^
Moderate	14 (13.5)	17 (23.9)	
Severe	7 (6.7)	16 (22.5)	

Comorbidity: number of comorbid chronic conditions; MMSE  =  Mini-Mental Status Examination; IADL  =  instrumental activities of daily living; GDS  =  Global Deterioration Scale;

a two-tailed T test;

b chi^2^ test;

c 11 missing values in each subsample;

d number of chronic comorbid conditions.

### Resource use

**Figure 2:**
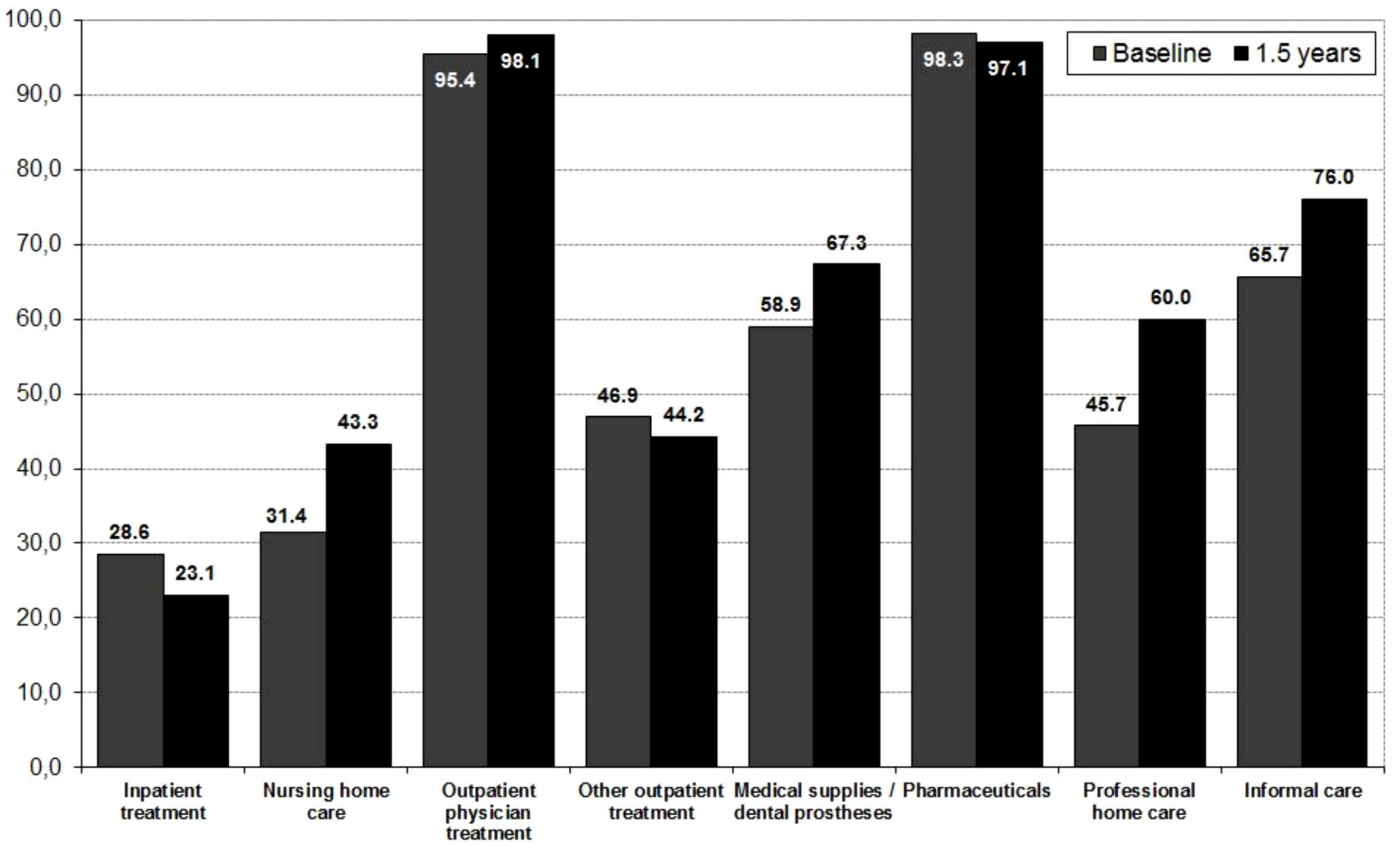
Sample proportions with resource use: baseline vs. 1.5 years.

Figure 2 shows the proportions of the dementia and control samples with resource use by healthcare sector at baseline and 1.5 years after baseline. Additionally, average amounts of resource use for selected services are presented in Table 4. At baseline, dementia patients required 2.2 hours or professional nursing care per week, while after 1.5 years, this figure had risen to 6.2 hours per week. However, no trend over time is evident in unadjusted values for use of medical care or informal care.

**Table 4:** Unadjusted resource use per capita.

Mean (SD)	Baseline (N = 175)	1.5 years (N = 104)
**Medical care**		
Days in hospital^a^	**3.4**	**3.3**
*(6 months)*	(7.8)	(9.9)
GP contacts	**1.7**	**1.2**
*(3 months)*	(3.0)	(2.5)
Specialist contacts	**1.6**	**1.5**
*(3 months)*	(2.6)	(2.4)
Number of pharmaceuticals	**6.6**	**7.0**
*(3 months)*	(4.0)	(3.9)
**Nursing care**		
Days in nursing home *(6 months)*	**45.4** (75.2)	**66.1** (85.7)
Professional home care *(hours/week)*	**2.2** (5.2)	**6.2** (16.0)
Informal care *(hours/week)*	**12.2** (21.5)	**12.5** (18.6)

a General and specialised psychiatric/neurological hospitals, excluding day patient treatment.

### Healthcare costs

Unadjusted average costs by healthcare sector across waves are presented in Table 5. Total 6-month costs in dementia patients rose from € 15,265 (US-$ 21,218) to € 17.073 (US-$ 23,731) over the 1.5-year interval. It is evident that a large proportion of total costs is due to the different areas of nursing care, with a trend towards an increasing share of nursing home costs over time.

**Table 5:** Unadjusted 6-month costs (€ 2008).

Mean *(SD)*	Baseline (N = 175)	0.5 years(N = 129)	1 year(N = 114)	1.5 years(N = 104)
Inpatient care	1,709 *(3,906)*	2,119 *(5,215)*	1,190 *(3,832)*	1,627 *(4,589)*
Outpatient physician care	384 *(521)*	392 *(454)*	349 *(356)*	343 *(369)*
Other outpatient treatment	*253 (515)*	280 *(582)*	170 *(425)*	236 *(458)*
Medical supplies and dentures	294 *(523)*	204 *(451)*	228 *(458)*	250 *(443)*
Pharmaceuticals	761 *(660)*	706 *(636)*	699 *(620)*	778 *(675)*
Medical care costs	3,400 *(4,408)*	3,700 *(5,491)*	2,636 *(4,122)*	3,234 *(4,857)*
Nursing home care	2,988 *(4,965)*	3,800 *(5,432)*	3,816 *(5,453)*	4,591 *(5,913)*
Professional home care	2,814 *(7,281)*	2,639 *(7,913)*	2,586 *(6,690)*	3,083 *(7,789)*
Informal care	5,939 *(10,472)*	7,366 *(10,372)*	7,461 *(12,321)*	6,087 *(9,034)*
Nursing care costs	11,741*(12,104)*	13,804 *(11,649)*	13,863 *(12,276)*	13,761 *(9,782)*
**Total costs**	**15,265 ** ***(13,143)***	**17,520 ** ***(13,308)***	**16,555 ** ***(12,933)***	**17,073 ** ***(12,025)***

Exchange rate: 1.39 US-$ per € (2008).

### Cost predictors in dementia

Table 6 presents the results of linear mixed models that analyse predictors of costs in dementia patients. There were highly significant effects of both ADL (Barthel score) and IADL deficits on total costs. Nursing home residence was associated with lower costs than being cared for in the community (€ 4,554 per six months, or US-$ 6,330). ADL deficits were the only significant predictor for medical care costs. With regard to formal nursing care, there were significant effects of age (€ 326 or US-$ 453 in six months per year of age), gender (additional 6-month costs of € 1.267 or US-$ 1,761 for male patients), nursing home residence (extra costs of € 6.469 or US-$ 8,992) and ADL deficits on costs. Finally, informal care costs declined with age (€ 280 or US-$ 389 in six months per year of age). They were influenced by comorbidity and IADL deficits, and, not surprisingly, informal care costs were substantially lower (€ 9,242 or US-$ 12,846 per six months) in nursing home residents.

**Table 6:** Longitudinal predictors of costs in dementia patients (6-month costs in € at 2008 values).

	Total costs	Medical care costs	Formal care costs	Informal care costs
Predictor variables	b(SE)	b(SE)	b(SE)	b(SE)
Time *(6-month intervals)*	- 120 (345)	- 269 (161)	148 (141)	65 (291)
Age *(centered)*	89 (125)	16 (42)	**326 (74)** ***	**- 280 (89)** **
Sex *(Ref.: female)*	923 (1,020)	- 123 (369)	**1,267 (521)** *	- 238 (813)
Comorbidity at baseline *(weighted count)*	73 (53)	- 7 (18)	- 38 (23)	**123 (44)** **
Nursing home residence *(Ref.: Living at home)*	**- 4,554 (1,364)** **	- 621 (675)	**6,469 (865)** ***	**- 9,242 (1,089)** ***
Barthel index *(centered)* a	**121 (32)** ***	**38 (12)** **	**44 (19)** *	14 (27)
IADL score *(centered)* a	**1,659 (354)** ***	264 (137)	156 (225)	**1,183 (306)** ***
GDS *(centered)*	208 (901)	- 406 (410)	537 (335)	340 (791)
Intercept	**9,983 (1,594)** ***	**3,161 (711)** ***	**2,294 (759)** **	**4,179 (1,357)** **
R^2^ within	0.07	0.09	0.31	0.02
R^2^ between	0.32	0.08	0.23	0.33
R^2^ overall	0.32	0.08	0.30	0.28
N	174	174	174	174

Linear mixed models with random effects for individuals;

*p <0,05;

**p<0,01;

***p<0,001;

SE  =  standard errors, based on nonparametric bootstrapping (4,000 replications);

a Barthel index and IADL score reverse coded. Exchange rate: 1.39 US-$ per € (2008).

### Sensitivity analysis

The results of the sensitivity analysis are presented in Table 7. When informal care is valued at the minimum wage rate of €10.96/hour, there is no significant effect of nursing home residence on total costs as compared to residence in the community. With informal care valued as lost leisure, nursing home residence is associated with lower total costs (€ 4,188 per six months, or US-$ 5,821), an effect which is even more pronounced if informal care is valued as lost production (€ 10,278 per six months, or US-$ 14,286).

**Table 7:** Sensitivity analysis: Longitudinal predictors of informal care costs and total costs in dementia patients (6-month costs in € at 2008 values) for different unit costs for informal care.

Approach	Replacement cost approach		Opportunity cost approach		Opportunity cost approach	
Unit cost: informal care	Minimum wage (€ 10.96/h)		Lost leisure (€ 18.00/h)		Lost production (€ 29.60/h)	
	Informal care	Total costs	Informal care	Total costs	Informal care	Total costs
Predictor variables	b(SE)	b(SE)	b(SE)	b(SE)	b(SE)	b(SE)
Time *(6-month intervals)*	38 (171)	- 159 (262)	62 (280)	- 123 (337)	102 (461)	- 82 (489)
Age *(centered)*	**- 164 (52)** **	199 (104)	**- 270 (85)** **	99 (122)	**- 444 (140)** **	- 72 (164)
Sex *(Ref.: female)*	- 139 (477)	1,007 (802)	- 229 (783)	932 (998)	- 377 (1,288)	763 (1,411)
Comorbidity at baseline *(weighted count)*	**72 (26)** **	21 (39)	**119 (43)** **	68 (51)	**195 (170)** **	147 (75)
Nursing home residence *(Ref.: Living at home)*	**- 5,420 (639)** ***	- 478 (1,133)	**- 8,901 (1,049)** ***	**- 4,188 (1,337)** **	**- 14,637 (1,725)** ***	**- 10,278 (1,868)** ***
Barthel index *(centered)* a	8 (16)	**113 (26)** ***	13 (26)	**120 (31)** ***	22 (43)	**130 (44)** **
IADL score *(centered)* a	**694 (180)** ***	**1,165 (283)** ***	**1,139 (295)** ***	**1,614 (347)** ***	**1,874 (485)** ***	**2,383 (496)** ***
GDS *(centered)*	199 (464)	146 (720)	327 (762)	197 (882)	538 (1,252)	429 (1,253)
Intercept	**2,451 (796)** **	**8,140 (1,298)** ***	**4,025 (1,307)** **	**9,828 (1,562)** ***	**6,618 (2,149)** **	**12,355 (2,185)** ***
R^2^ within	0.02	0.13	0.02	0.08	0.02	0.04
R^2^ between	0.33	0.31	0.33	0.32	0.33	0.34
R^2^ overall	0.28	0.34	0.28	0.32	0.28	0.31
N	174	174	174	174	174	174

Linear mixed models with random effects for individuals;

*p<0,05;

**p<0,01;

***p<0,001;

SE  =  standard errors, based on nonparametric bootstrapping (4,000 replications);

a Barthel index and IADL score reverse coded. Exchange rate: 1.39 US-$ per € (2008).

## Discussion

Our results show that total costs in dementia patients are mostly determined by impairment in basic and instrumental activities of daily living. Also, there was a substantial negative effect of nursing home residence on overall costs, indicating that the societal costs of caring for patients in the community can be considerably higher than nursing home costs if informal care is taken into account. This is also reflected in the effects of nursing home residence on formal and informal care, respectively, with the negative effect of nursing home residence on informal care costs exceeding the positive effect of nursing home residence on formal care costs. With respect to separate cost categories, ADL deficits were associated with higher medical and formal care costs, but not informal care costs. The latter, by contrast, were significantly increased by IADL deficits. This split between ADL and IADL deficits as determinants of formal care on the one and informal care on the other hand might indicate that ADL deficits are predominantly met by formal care, while assistance with IADL is a domain covered by informal caregivers. There was no overall effect of age, gender or comorbidity. However, formal care costs increased with age and were higher in men than in women, while controlling for living situation. This gender effect might be linked to the fact that men in this age range are more likely to be living with a spouse than women. In our sample, slightly more than half of the men were living with their spouse at both baseline and at 1.5 years, as opposed to fewer than 20% of the women (data not shown). Men might thus be able to remain in their homes, being cared for by their spouses and using formal care in addition, in a situation in which women in the absence of a spouse, who is able to provide informal care and/or arrange for professional home care, might have to be admitted to a nursing home. Comorbidity was associated with higher informal care costs only. In particular, there was no effect of comorbidity on medical care costs. Of note, we found no effect of cognitive impairment as measured by the GDS on either total costs or costs in any of the subcategories. This implies that cognitive impairment, which is itself the cause of functional impairment in dementia, is associated with need for care and corresponding costs only via its effect on the patients' functional capacity as reflected in the ADL and IADL score. The costs of informal care, in particular, depend on the method of valuation. We therefore varied the unit costs for informal care, using three separate scenarios. On the whole, this sensitivity analysis supports the conclusion that – from a societal point of view – nursing home care is associated with lower total costs than care in the community. At one extreme in our sensitivity analysis, if informal care is valued at a minimum wage rate under the replacement cost approach, there is no significant cost difference in total costs between nursing home residence and community residence. However, if informal care is valued in terms of lost leisure or, at the other extreme, in terms of lost production under the opportunity cost approach, care in the community is associated with additional costs of approximately € 4,000 or € 10,000 per six months (US-$ 5,500 or US-$ 14,000).

### Previous longitudinal COI studies

Overall, our results are in good accordance with previously published longitudinal COI studies in dementia. Across studies, functional decline emerges as the principal predictor of costs of care in dementia. Andersen et al. [9] reported such an effect in a longitudinal study of costs of care from a societal perspective which did not, however, include informal care. In this study, the move into a nursing home was associated with a marked increase in total costs. Similarly, in another study of formal care costs only Zhu et al. [12] found living at home to be associated with significantly lower costs. Again, costs were significantly determined by functional impairment, with another significant effect of comorbidity. These findings correspond to our results; however, in our sample, the additional formal care costs associated with nursing home residence are more than counterbalanced by the decreased informal care costs. As to determinants of informal care costs results are essentially similar, with significant effects of functional impairment reported in studies by Zhu et al. [10], [11] and Rapp et al. [13]. Zhu et al. [10] also reported an effect of patient dependence on other individuals in addition to the effect of functional impairment. There is little evidence that cognitive impairment as such has an effect on costs beyond the effect of functional impairment (which is itself a consequence of cognitive deficits). Rapp et al. [13] do find an effect of MMSE on costs, but report that this effect is diminished when ADL is simultaneously included in the analysis. Comorbidity was shown in different studies to have effects on costs of medical care [10], formal care costs [9], [12] and use of informal care [11]. Gender effects were not consistently found and varied in direction.

### Strengths and limitations

A major strength of the AgeCoDe study is that the cohort was recruited via GP offices. Since 93% of people aged 70 or older regularly visit a GP, the cohort can be regarded as close to representative for this age bracket, even though a degree of participation bias cannot be ruled out [14]. With respect to disease severity in the dementia subsample, the proportions are likely to be biased towards the mild stage in comparison with the general population of the same age, due to the exclusion of subjects with dementia at recruitment, i.e. those subjects who had developed dementia before the age of 75. Also, as the baseline differences between dementia patients with complete data over 1.5 years and drop-outs after the baseline assessment indicate, the more severely demented patients at baseline may have been disproportionately more likely to drop out during the course of the study. Consequently, the longitudinal data analysed here may not fully reflect the natural course of dementia. However, while these limitations may lead to unadjusted total costs that underestimate costs of dementia, they should not affect the results of the regression analyses presented in this study, in which dementia severity or indicators of impairment are controlled for. Another issue to be kept in mind is that subjects were at least 79 years old at baseline. This is a strength insofar as there are few COI studies in dementia which have investigated samples of a similarly advanced age. On the other hand, however, patterns of care and resulting cost estimates might differ in populations with a more varied age structure.

With regard to the valuation of informal care, one caveat concerns the assumption inherent in the replacement cost approach (or proxy good method) that professional care and informal care are substitutes, i.e. that informal care perfectly replaces formal care. It may be argued that professional caregivers are likely to be more efficient at their tasks than informal caregivers, so that valuing informal care time using hourly wage rates for professional caregivers overestimates the costs of nursing care. However, the sensitivity analysis demonstrates that using the opportunity cost approach as an alternative for the valuation of informal care either has very little effect on costs, if informal care time is regarded as lost leisure and an hourly rate of €18.00 is applied, or is associated with even higher estimates for costs of informal care, if informal care time is assumed to constitute lost production and is valued at € 29.60 per hour.

### Conclusions

This is one of few longitudinal studies so far to examine overall COI in dementia from a societal perspective, including formal as well as informal care. Findings confirm the role of ADL and IADL impairment as the principal cost determinants. Unlike similar previous studies the analyses presented here afford estimates of the effect of nursing home residence on costs of formal nursing care and informal care as well as on total costs from a societal perspective. Findings indicate that, although cost estimates vary depending on the method of valuation for informal care, care for patients who live in the community is associated with higher total costs than nursing home residence. The fact that from a societal perspective nursing home residence is associated with relatively smaller costs than care in a community setting does not imply that nursing home care should necessarily be preferred, but it underlines the societal relevance that informal care has within the larger current framework of care for dementia patients.

## Supporting Information

Table S1 Detailed unit costs (base case analysis).
(DOCX)
