# Supplementary material for: Predictors of Costs in Dementia in a Longitudinal Perspective
Source: PLoS One. 2013 Jul 18;8(7):e70018. doi: 10.1371/journal.pone.0070018 (PMC3715502; doi:10.1371/journal.pone.0070018)
Supplement: Table S1 — Detailed unit costs (base case analysis). (DOCX) [file pone.0070018.s001.docx]

| **S1. Detailed unit costs** (base case analysis). | | | | |
| --- | --- | --- | --- | --- |
|  | Cost (year 2008 values) | | | Source |
|  | | | | |
| **Inpatient treatment:** costs per day | | | | |
| General hospital | *Inpatient treatment* | | € 483.57 | Federal Statistical Office, German Hospital Federation, Statutory Pension Insurance Fund [33-35] |
|  | *Day-patient treatment* | | € 314.32 |  |
| Psychiatric hospital | *Inpatient treatment* | | € 251.75 |  |
|  | *Day-patient treatment* | | € 163.64 |  |
| Rehabilitation clinic | *Inpatient treatment* | | € 105.31 |  |
|  | *Day-patient treatment* | | € 68.45 |  |
|  |  | |  |  |
| **Outpatient physician treatment:** costs per contact | | | | |
| GP | | | € 17.77 | Calculated costs per contact [36] |
| Cardiologist | | | € 68.52 |  |
| Neurologist | | | € 14.60 |  |
| Psychiatrist | | | € 14.60 |  |
| Orthopaedist | | | € 22.28 |  |
| Ear, nose and throat specialist | | | € 23.65 |  |
| Dermatologist | | | € 14.86 |  |
| Ophthalmologist | | | € 24.87 |  |
| Gynaecologist | | | € 25.56 |  |
| Urologist | | | € 27.46 |  |
| Surgeon | | | € 21.81 |  |
| Radiologist | | | € 79.43 |  |
| Psychotherapist | | | € 45.17 |  |
| Dentist | | | € 40.09 |  |
| Casualty department | | | € 36.03 |  |
|  |  | |  |  |
| **Other outpatient treatment:** costs per contact | | | | |
| Physiotherapy | | | € 15.78 | Reimbursement schedules (Statutory health insurance funds [37-39]), calculated costs per contact [36] |
| Occupational therapy | | | € 32.23 |  |
| Speech therapy | | | € 21.22 |  |
| Massage therapy / Lymphatic drainage | | | € 16.71 |  |
| Podiatry | | | € 25.18 |  |
|  |  | |  |  |
| **Medical supplies and dental prostheses:** costs per item | | | | |
| Surgical stocking |  | | € 42.45 | Reimbursement schedules (Statutory health insurance funds [40]) |
| Hearing aid |  | | € 421.28 |  |
| Wheelchair |  | | € 604.61 |  |
| Walking stick |  | | € 36.13 |  |
| Pair of spectacles |  | | € 27.64 |  |
| Bandages (package) |  | | € 3.04 |  |
| Incontinence pads |  | | € 0.19 |  |
| Tooth crown |  | | € 239.16 | Reimbursement schedules (Federal Association of Panel Dentists [41]) |
| Pontic |  | | € 566.95 |  |
| Dental prosthesis |  | | € 543.64 |  |
|  |  | |  |  |
| **Pharmaceuticals** |  | | |  |
| Specific products | | Retail prices according to: | | *Rote Liste 2008* [42] |
|  | | | | |
| **Nursing home care:** costs per day | | | | |
| Residential care | *Care level 1* | | € 56.40 | Calculated costs of care per day (Federal Statistical Office [26]) |
|  | *Care level 2* | | € 70.76 |  |
|  | *Care level 3* | | € 85.13 |  |
| Day care | *Care level 1* | | € 40.00 |  |
|  | *Care level 2* | | € 45.64 |  |
|  | *Care level 3* | | € 52.31 |  |
|  |  | |  |  |
| **Professional home care:** costs per hour | | | € 18.69 | Hourly gross wage rate plus non-wage labour costs for employees in the domain of care and assistance for the elderly or handicapped (Federal Statistical Office [43,44]) |
|  |  | |  |  |
| **Informal care:** costs per hour | | | € 18.69 | *Replacement cost method:* see professional home care |
|  |  | |  |  |
|  |  | | |  |
